# Optimization of culture condition for *Spodoptera frugiperda* by design of experiment approach and evaluation of its effect on the expression of hemagglutinin protein of influenza virus

**DOI:** 10.1371/journal.pone.0308547

**Published:** 2024-08-16

**Authors:** Fatemeh Alizadeh, Hamideh Aghajani, Fereidoun Mahboudi, Yeganeh Talebkhan, Ehsan Arefian, Sepideh Samavat, Rouhollah Raufi

**Affiliations:** 1 Biotechnology Research Center, Department of Medical Biotechnology, Pasteur Institute of Iran, Tehran, Iran; 2 Department of Research & Development, AryoGen Pharmed Inc., Karaj, Iran; 3 Molecular Virology Lab, Department of Microbiology, School of Biology, College of Science, University of Tehran, Tehran, Iran; Cairo University Faculty of Veterinary Medicine, EGYPT

## Abstract

The baculovirus expression vector system (BEVS) is a powerful tool in pharmaceutical biotechnology to infect insect cells and produce the recombinant proteins of interest. It has been well documented that optimizing the culture condition and its supplementation through designed experiments is critical for maximum protein production. In this study, besides physicochemical parameters including incubation temperature, cell count of infection, multiplicity of infection, and feeding percentage, potential supplementary factors such as cholesterol, polyamine, galactose, pluronic-F68, glucose, L-glutamine, and ZnSO_4_ were screened for *Spodoptera frugiperda* (Sf9) cell culture and expression of hemagglutinin (HA) protein of Influenza virus via Placket-Burman design and then optimized through Box-Behnken approach. The optimized conditions were then applied for scale-up culture and the expressed r-HA protein was characterized. Optimization of selected parameters via the Box-Behnken approach indicated that feed percentage, cell count, and multiplicity of infection are the main parameters affecting r-HA expression level and potency compared to the previously established culture condition. This study demonstrated the effectiveness of designing experiments to select and optimize important parameters that potentially affect Sf9 cell culture, r-HA expression, and its potency in the BEVS system.

## Introduction

Recombinant proteins produced in prokaryotic (mostly bacteria) and eukaryotic (fungi, insect, and mammalian) expression systems are used as vaccines and therapeutics [1]. Since 1983, baculovirus expression vector systems (BEVS) within insect cells have been widely used to produce recombinant proteins, designation of viral vectors for gene therapy and gene delivery, and antigen carriage [2]. These viruses are the most prominent double-stranded, circular DNA ones that infect insect cells [3, 4] mainly Lepidopteran cell lines including *S*. *frugiperda* (Sf21 and Sf9), and *Trichoplusia ni* (HighFiveTM) which are the commonly used cells in suspension cultures [5].

The advantages of BEVS such as high expression level, ease of scale-up, adaptability to suspension culture, and acceptable post-translational modifications (PTMs) make it a powerful system for the production of recombinant proteins [6, 7]. Well-characterized products originating from these systems include CERVARIX® (against cervical cancer), PROVENGE® (against prostate cancer), FluBlok® (influenza vaccine), and Nuvaxovid® (covid-19 vaccine) [8].

According to the World Health Organization (WHO) reports, annual influenza occurrence is one of the most important epidemics that involves approximately 5–15% of the Northern Hemisphere population and causes about 290,000 to 650,000 global respiratory deaths indicating vaccination is the most efficient preventive approach [9]. Amongst four types of influenza virus (A, B, C, and D), types A and B are usually included in annual influenza vaccines. In comparison, the other two types have not represented significant impacts on human population health [10]. Influenza type A has more than 100 subtypes based on random mutations that usually occur within its two surface proteins, hemagglutinin (HA) and neuraminidase (NA) which the former is a spike-shaped protein that sticks to the viral surface [10]. Therefore, influenza (flu) vaccines need to be updated annually [10]. Unlike traditional egg-based flu vaccines which encounter disadvantages such as pathogen-free egg shortage, complex purification procedures, egg-based protein impurities, antibiotics, and preservatives within the final product [9], the BEVS-based recombinant flu vaccines compromise HA gene fragment replaced with polyhedrin gene and expressed under the control of its promoter within virally infected insect cells [11–13] that has no infective live virus particle and is free from adjuvants, antibiotics, preservatives, possible pathogens, and unrelated proteins [9].

Common basic media for insect cell culture are usually chemically defined and consist of amino acids, sugars, vitamins, organic acids, and inorganic salts [14] which their selection and screening are critical in any process development [15]. Therefore, plenty of time and budget is usually paid for media selection and optimization of culture conditions [16, 17] through the design of experiments (DOE) [18, 19]. DOE is a statistical approach for identifying effective parameters and their optimal levels through defined experiments to lower costs and increase efficiency [20]. Several DOE approaches (Full and fractional factorial) are designated based on the number of selected parameters, their defined levels, and the time and budget dedicated to each study [20].

Increasing the HA potency through optimization procedures inevitably reduces the industrial production scale and costs which are great achievements for the healthcare systems. Therefore, in the present study, potentially effective Sf9 culture parameters/supplements were selected by a literature review which include feed percentage, cell count of infection (CCI), multiplicity of infection (MOI), temperature, cholesterol, polyamine, galactose, pluronic-F68, initial cell density, glucose, L-glutamine and ZnSO_4_ [10, 20–34]. In the next step, their impacts on r-HA expression were screened using DOE and response surface methodology (RSM) to reduce the cost and time of experiments.

## Materials and methods

### Cell culture and viral infection

Sf9 cells (OET, UK) were cultured in 125 ml shake flasks containing 30 ml PSFM-J1 medium (Fujifilm, Japan) and incubated at 28°C, 85 rpm (pH 6.0–6.4; osmolality of 345–380 mOsm/kg) with an initial cell density of 1.5–2×10^6^ cells/ml. The cells were sub-cultured when viability and cell density reached ≥90% and 4–5×10^6^ cells/ml, respectively. After 5 passages, viral infection was done based on the desired CCI (cells/ml): MOI (PFU/ml) ratio. After 48 to 96 hours when the viability dropped to 40–60%, the cells were harvested by centrifugation at 4,000 rpm for 15 min.

### Design of experiment by Plackett-Burman approach

Selected parameters for optimization of insect cell culture were initial cell density (0.8–2×10^6^ cells/ml), feed percentage (3–9% of the total culture volume), CCI (2–6×10^6^ cells/ml), MOI (0.3–3 PFU/ml), temperature (22–28°C), cholesterol (4–20 mg/L), polyamine (0.5–1.5X), galactose (10–30 mM), pluronic-F68 (0.1–0.5 w/v), glucose (10–20 g/L), L-glutamine (10–20 mM), and ZnSO_4_ (10–40 μM). All selected parameters were screened by Plackett-Burman design in Design Expert® (v.11) in 23 experiments at 2 (low and high) levels (S1 Table) and their effects on HA expression level (the main objective), cell density, and cell viability were daily monitored within 500 ml baffled shake flasks with a working volume of 100 ml. The cells were harvested by centrifugation at 4,000 rpm for 15 min when the viability dropped to 40–60%. The optimization procedure was conducted in comparison to the previously developed un-supplemented culture condition in which a defined constant CCI: MOI ratio (6.0 ×10^6^ cells/ml: 0.5 PFU/ml) was applied for viral infection after 72h incubation and the cells were harvested at 40–60% viability.

### Evaluation of protein expression

The harvested cells were resuspended in lysis buffer (20 mM Na_2_CO_3_, 30 mM NaHCO_3_, 0.1% 2ME, 50 mM NaCl, pH 9.0±0.3) and incubated at 2–8°C for 40 min on a shaker followed by centrifugation at 9,000 rpm for 15 min at room temperature (RT). The pellet was resuspended in extraction buffer (10 mM ethanolamine, 0.2% Triton X-100, 0.1% 2ME, 25 mM NaCl, pH 7.0±0.3) and incubated on a shaker for a further 20 h (RT). After centrifugation at 9,000 rpm for 15 min, the supernatant was filtered (0.22 μm) and run on a gradient (4, 6, 8, 12, 15, 20%) SDS-PAGE. The intensity of the r-HA protein band was evaluated using Image Lab® software (Bio-Rad, USA) to estimate its approximate protein concentration based on the standard HA protein band (Flublok, Sanofi) according to the following formula:

HAconcentration(μg/ml)=[DensityoftheHAband(OD)×StandardHAconcentration(μg/ml)]/DensityofstandardHA(OD)

### Response surface methodology

In the next step, the optimization of significantly effective parameters was performed using Box-Behnken response surface methodology in three levels (low, middle, and high) by Design Expert. Fifty-four experiments were run (S2 Table). Cell culture procedure, protein extraction, and expression evaluation were done according to the mentioned protocols. Cell viability and HA expression level were evaluated as the main responses.

### Bioreactor culture

Based on DOE experiments, the optimal conditions were applied to benchtop 2 L bioreactors (Eppendorf, Germany) where the main responses were monitored compared to the previously described established control culture condition. In brief, the insect cells were expanded for up to 4 passages and transferred into three 2 L benchtop bioreactors (working volume of 1700 ml) under control and optimized conditions. After 72 h, the cells were infected with optimized infection condition and incubated for protein expression till the viability dropped to 40–60%.

### r-HA protein purification

The soluble protein fraction was extracted from harvested cells and filtered as previously described. The filtered protein solution was loaded on DEAE-S resin (Arg Biotech, Iran) where r-HA was separated in a flow-through mode. The diluted r-HA containing flow-through sample was loaded on Hi-Trap Capto Lentil Lectin affinity chromatography resin (Cytiva, USA) within the equilibration buffer (30 mM Tris, 500 mM NaCl, 0.05% Triton X-100, 0.01% 2-ME, pH 8.3, conductivity of 4.7 ms/cm) and eluted by the elution buffer (30 mM Tris, 200 mM D-glucose, 0.003% Tween-20, 0.03% Triton X-100, 150 mM NaCl, pH 7.4, conductivity of 14 ms/cm), buffer exchanged in phosphate buffer (7.3 mM Na_2_HPO_4_, 2.8 mM NaH_2_PO_4_, 150 mM NaCl) by diafiltration and its concentration was measured by BCA method.

### Potency measurement

Single Radial Immunodiffusion assay (SRID) has been internationally recognized as the gold standard method in the potency assessment of HA protein. The assay is based on the reaction of HA and its specific antibody in which HA concentration can be proportionally calculated by the size of the ring (diameter) relative to the known standard HA protein. In this assay, standard HA protein (Strain 2-Influenza Antigen-B-Phuket-3073-2013-NIBSC, UK; 66 to 0.515 μg/ml concentrations) and purified r-HA (1:1 to 1:128 dilutions) were loaded into wells of agarose gel supplemented with annually generated anti-HA antibodies. After 18–20 h incubation at 25°C, the wells were stained with coomassie blue and the produced immune-precipitin rings were quantified with Digimizer® software.

### Protein characterization

The purified r-HA protein was characterized compared to the standard HA protein. The characterization tests included purity, native folding, glycan profiling, and size heterogeneity. Recombinant HA characteristics were compared to the original protein through standard and homemade assays.

### Statistical analysis

The obtained data has been presented as the mean of three experiments with standard deviation (SD) after evaluation of data normalization with the Shapiro-Wilk normality test. The mean values were compared using the student’s t-test. ANOVA analysis was applied in optimization studies. The significant differences were considered to be 0.05. Figures were created using GraphPad Prism (v.8.0) and Design Expert (v.11.0).

## Results

### Screening of culture conditions by Plackett-Burman design

**Fig 1:**
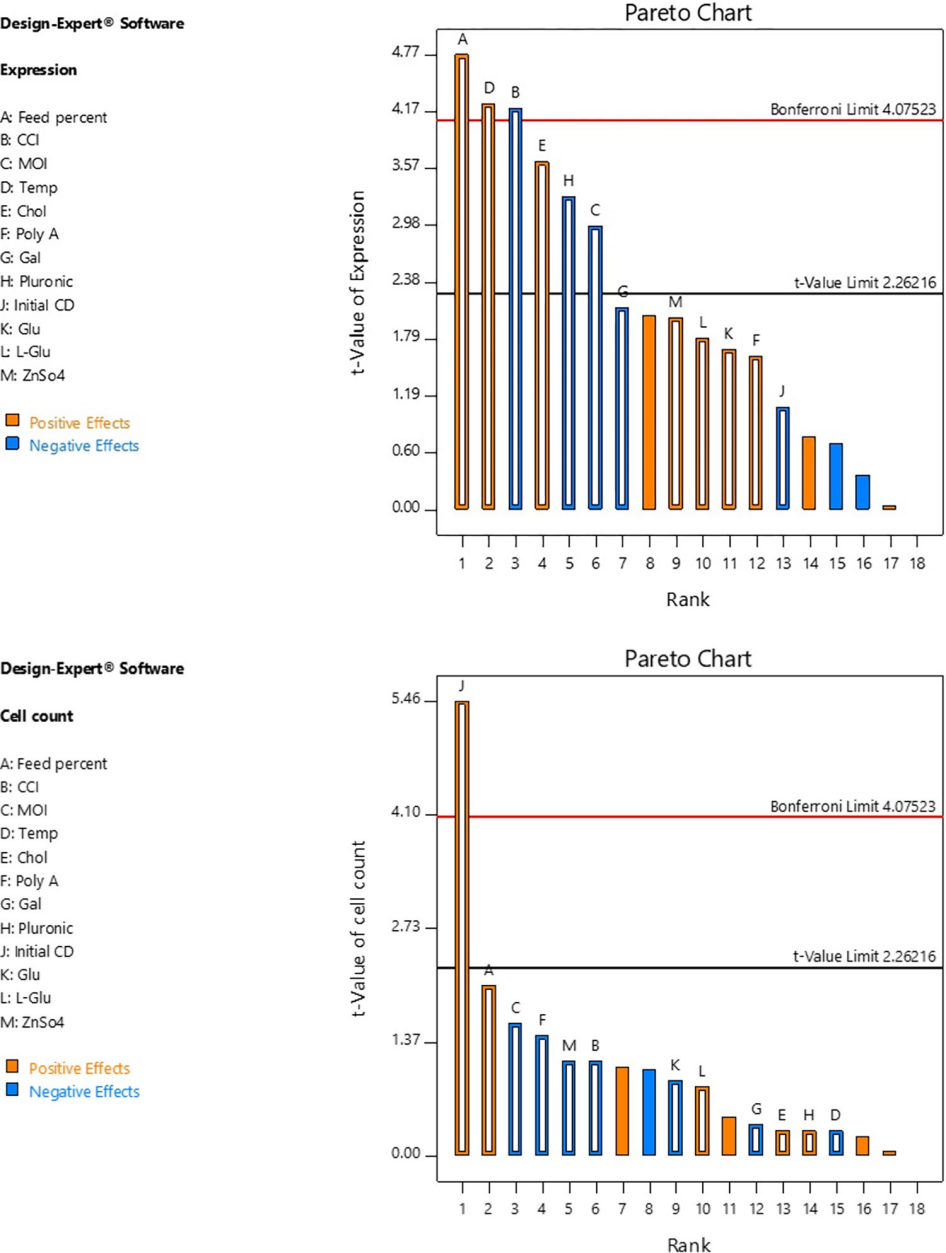
Pareto charts for the Plackett-Burman design. The charts demonstrate the order and effect of each parameter on **(A)** r-HA expression level and **(B)** Viable cell count.

Plackett-Burman design was applied to screen selected parameters and study their effects on cell density and HA expression level. Other responses such as cell viability were also analyzed. Amongst the studied parameters, six parameters had significant effects on r-HA expression level, while only one affected cell density (Fig 1 and S3 Table).

The ANOVA results summarized in Table 1 suggested the initial cell density as the only effective positive parameter for cell count response. Feed percentage, temperature, and cholesterol had positive effects on HA expression level, while CCI, MOI, and pluronic represented significantly negative effects (Fig 1).

**Table 1:** Variance analysis of the selected parameters in Placket-Burman-designed experiments.

Source	df	Viable cell count (×10^6^ cells/ml)				Expression level (μg/ml)			
		Sum of Squares	Mean Square	F value	*P* value	Sum of Squares	Mean Square	F value	*P* value
Model	12	15.00	1.25	4.11	0.0266	13541.28	1128.44	10.36	0.0013
A, Feed percentage	1	1.28	1.28	4.20	0.0747	2472.23	2472.23	22.71	**0.0014**
B, CCI	1	0.3937	0.3937	1.30	0.2880	1924.13	1924.13	17.67	**0.0030**
C, MOI	1	0.7718	0.7718	2.54	0.1498	962.00	962.00	8.84	**0.0178**
D, Temperature	1	0.0280	0.0280	0.0921	0.7693	1971.60	1971.60	18.11	**0.0028**
E, Cholesterol	1	0.0280	0.0280	0.0921	0.7693	1444.63	1444.63	13.27	**0.0066**
F, Polyamine	1	0.6317	0.6317	2.08	0.1874	282.00	282.00	2.59	0.1462
G, Galactose	1	0.0437	0.0437	0.1439	0.7143	488.93	488.93	4.49	0.0669
H, Pluronic	1	0.0280	0.0280	0.0921	0.7693	1172.60	1172.60	10.77	**0.0112**
J, Initial cell density	1	9.07	9.07	29.84	**0.0006**	126.23	126.23	1.16	0.3130
K, Glucose	1	0.2520	0.2520	0.8289	0.3892	306.60	306.60	2.82	0.1318
L, Glutamine	1	0.2117	0.2117	0.6965	0.4282	352.13	352.13	3.23	0.1098
M, ZnSO_4_	1	0.3937	0.3937	1.30	0.2880	440.00	440.00	4.04	0.0792
Residual error	8	2.43	0.3040			870.97	108.87		
Lack-of-Fit	6	2.29	0.3820	5.46	0.1629	748.97	124.83	2.05	0.3641
Pure Error	2	0.1400	0.0700			122.00	61.00		
Cor. total	21	19.11				29405.32			

Bolded values represent statistically significant parameters (P<0.05).

Viable cell count and HA expression level were expressed as empirical first-order polynomial equations in Eqs 1 and 2 where A, B, C, D, E, H, and J represent feed percentage, CCI, MOI, temperature, cholesterol, pluronic, and initial cell density, respectively.


$$Viable\;cell\;count(\times 10^{6}cells/ml):+6.29+0.72J$$ (Eq 1)

HAexpressionlevel(μg/ml):+93.49+11.89A‐10.49B‐7.41C+10.61D+9.09E‐8.19H (Eq 2)

**Fig 2:**
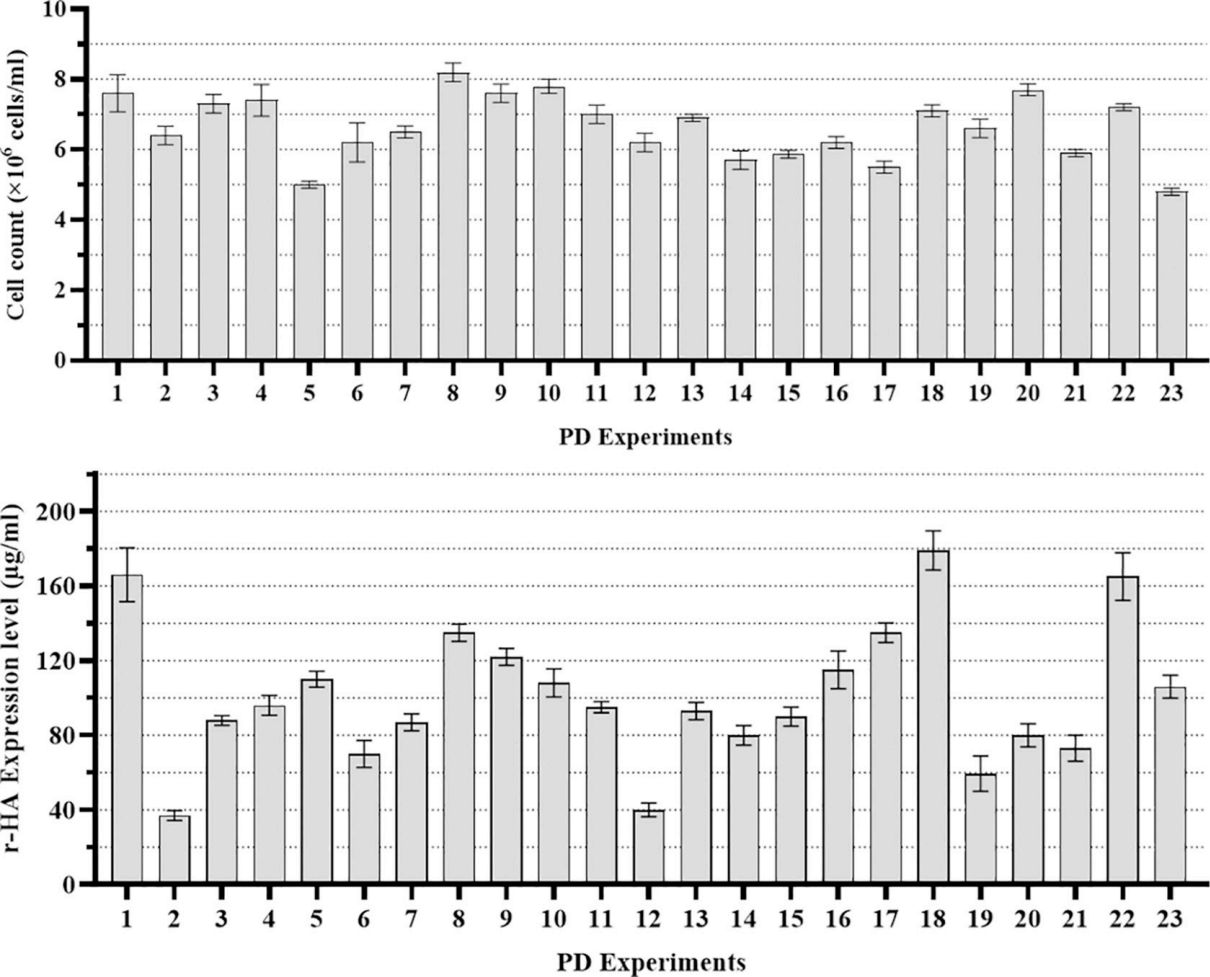
The comparative analysis of 23 Placket-Burman-designed experiments on main responses. (A) Cell count and (B) r-HA expression. Error bars represent the SD of three independent experiments.

Fig 2 shows the comparative analysis of 23 Placket-Burman-designed experiments on cell count and HA expression level as the main responses which confirms the ANOVA results.

### Optimization of culture condition by RSM

**Fig 3:**
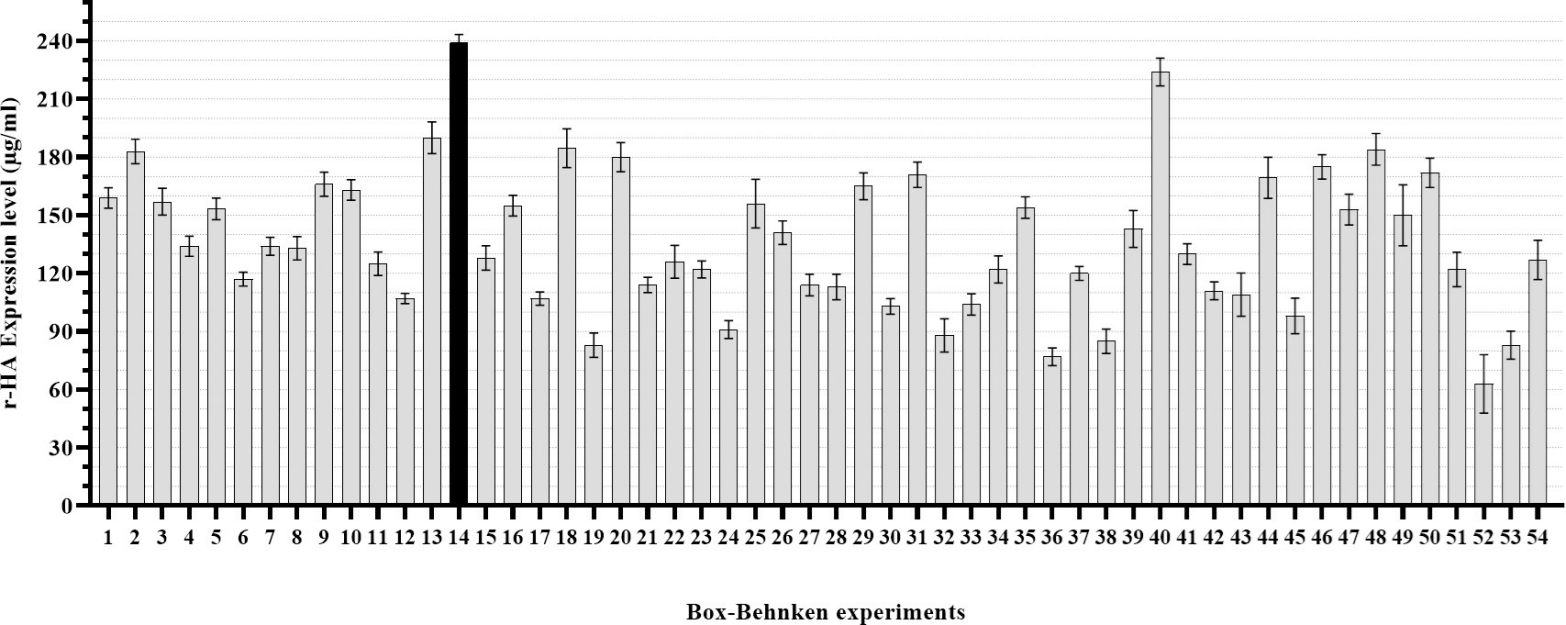
Comparative evaluation of HA expression level within 54 designed RSM experiments. The highest expression level has been achieved under conditions described in experiment#14 (Black bar). Error bars represent the SD of three independent experiments.

The significantly effective parameters selected from Plackett-Burman experiments (feed percentage, CCI, MOI, temperature, cholesterol, and pluronic) were further investigated in three levels within 54 experiments by response surface methodology (Box-Behnken) (S2 Table) amongst the feed percentage, CCI, and MOI were defined as statistically significant parameters (Table 2). Analysis of r-HA expression level represented a significant difference in experiment 14 (Fig 3). Other responses including viable cell count and viability were also monitored (S4 Table). As previously mentioned, the cells were intended to be harvested with 40–60% viability on day 7. However, in some experiments, the cells were harvested at earlier times due to their low cell count and viability.

**Table 2:** Analysis of variance for the effect of selected parameters from Box-Behnken designed experiments.

Source	Sum of Squares	df	Mean Square	F-value	*P*-value
Block	0.1716	1	0.1716		
**Model**	3.98	48	0.0829	104.82	**0.0002**
A, Feed percentage	0.0333	1	0.0333	42.07	**0.0029**
B, TOI	0.1072	1	0.1072	135.54	**0.0003**
C, MOI	0.0303	1	0.0303	38.28	**0.0035**
D, Temperature	0.0022	1	0.0022	2.77	0.1715
E, Cholesterol	0.0041	1	0.0041	5.24	0.0839
F, Pluronic	0.0051	1	0.0051	6.49	0.0635
AB	0.0007	1	0.0007	0.9369	0.3879
AC	0.0428	1	0.0428	54.10	**0.0018**
AD	0.0005	1	0.0005	0.5923	0.4845
AE	0.0314	1	0.0314	39.74	**0.0032**
AF	0.0022	1	0.0022	2.73	0.1739
BC	0.0241	1	0.0241	30.45	**0.0053**
BD	0.0010	1	0.0010	1.32	0.3152
BE	0.0159	1	0.0159	20.16	**0.0109**
BF	0.0213	1	0.0213	26.98	**0.0065**
CD	0.0030	1	0.0030	3.79	0.1233
CE	0.0011	1	0.0011	1.41	0.3009
CF	0.0095	1	0.0095	12.02	**0.0256**
DE	0.0438	1	0.0438	55.36	**0.0017**
DF	0.0001	1	0.0001	0.1687	0.7023
EF	0.0001	1	0.0001	0.1127	0.7539
A^2^	0.0299	1	0.0299	37.78	**0.0036**
B^2^	1.60	1	1.60	2019.78	**< 0.0001**
C^2^	0.0025	1	0.0025	3.12	0.1520
D^2^	0.1743	1	0.1743	220.40	**0.0001**
E^2^	0.1094	1	0.1094	138.31	**0.0003**
F^2^	0.0076	1	0.0076	9.61	**0.0362**
ABD	0.0256	1	0.0256	32.44	**0.0047**
ACF	0.0037	1	0.0037	4.70	0.0960
ADE	0.0002	1	0.0002	0.1928	0.6833
BCE	0.0624	1	0.0624	78.97	**0.0009**
BEF	0.0002	1	0.0002	0.2668	0.6327
CDF	0.0084	1	0.0084	10.59	**0.0312**
A^2^B	0.1074	1	0.1074	135.86	**0.0003**
A^2^C	0.0001	1	0.0001	0.0708	0.8033
A^2^D	0.0186	1	0.0186	23.48	**0.0084**
A^2^E	0.0004	1	0.0004	0.5199	0.5108
A^2^F	0.0001	1	0.0001	0.1542	0.7146
AB^2^	0.0024	1	0.0024	3.01	0.1578
AC^2^	0.0742	1	0.0742	93.82	**0.0006**
B^2^C	0.0139	1	0.0139	17.52	**0.0139**
B^2^D	0.0977	1	0.0977	123.51	**0.0004**
B^2^F	0.0161	1	0.0161	20.32	**0.0108**
BC^2^	0.0002	1	0.0002	0.2741	0.6283
C^2^E	0.0180	1	0.0180	22.75	**0.0088**
A^2^CF	0.0001	1	0.0001	0.1134	0.7532
AB^2^D	0.1580	1	0.1580	199.87	**0.0001**
BC^2^E	0.0003	1	0.0003	0.3587	0.5815
Pure Error	0.0032	4	0.0008		
Cor Total	4.15	53			

Bolded values represent statistically significant parameters (P<0.05).

**Fig 4:**
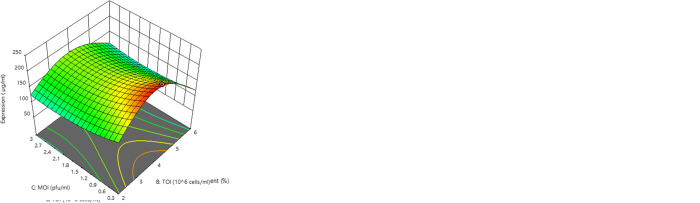
Response surface 3D plots represent the interactions between r-HA expression level and **(A)** Feed percentage and TOI; **(B)** Feed percentage and MOI; **(C)** CCI and MOI.

**Fig 5:**
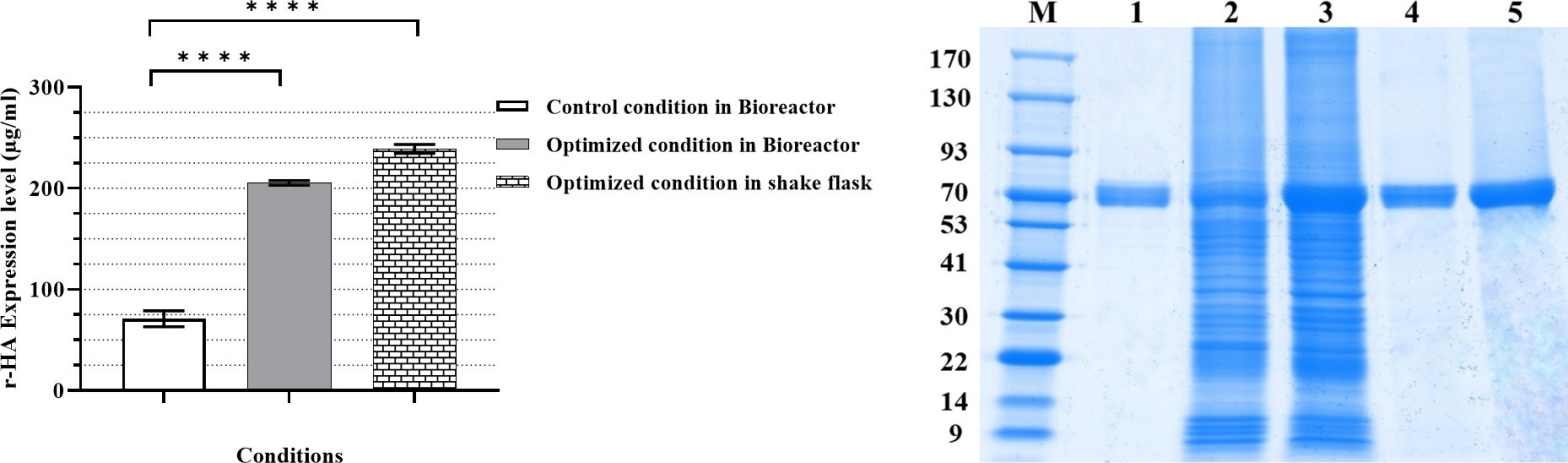
Evaluation of HA expression. **(A)** r-HA expression level in different culture conditions (Control condition in bioreactor, optimized condition in shake flask, and optimized condition in bioreactor: 71, 239, and 205 μg/ml, respectively); **(B) SDS-PAGE (non-reduced samples):** M: Mw marker; #1: Standard HA protein; #2: Cell lysate from control culture condition; #3: Cell lysate from optimized culture condition; #4: Eluate of lectin resin from control culture condition; #5: Eluate of lectin resin from the optimized condition. **** represents *P* <0.0001. Error bars show the SD of triplicate runs.

The modified quartic model describing the correlation of the variables and r-HA expression level has been presented in Eq 3 and Fig 4.

$$HA\;expression\;level(\mu g/ml):+5.20+0.06A+0.11B‐0.06C‐0.0731AC+0.0549BC‐0.05A^{2}‐0.39B^{2}‐0.16A^{2}B+0.13AC^{2}+0.05B^{2}C$$ (Eq 3)

where A, B, and C represent feed percentage, CCI, and MOI, respectively. According to the obtained results, the optimum condition for the highest r-HA expression level includes 5.37% feed, CCI of 5.32×10^6^ cells/ml, MOI of 1.65 PFU/ml, 12 mg/L of cholesterol, 0.35% of pluronic at 23.31°C. It seems that the optimization procedure carried out in this study effectively enhanced the expression level of the r-HA (Fig 5A)

### Scale-up of the optimized culture condition

To validate the obtained optimal condition from RSM experiments, fed-batch mode 2 L benchtop bioreactors were run under optimized condition (5.37% feed, CCI of 5.32×10^6^ cells/ml, MOI of 1.65 PFU/ml, 12 mg/L cholesterol, 0.35% pluronic at 23.31°C) when the control batches were run without any supplementation under CCI/MOI ratio of 6/0.5 at 28°C. The cells were harvested and the r-HA protein was purified as previously described (Fig 5B). The obtained r-HA expression level confirmed the reproducibility of the RSM results which was significantly elevated (3.6 folds) compared to the control culture condition (Fig 5A).

### Potency assay

**Fig 6:**
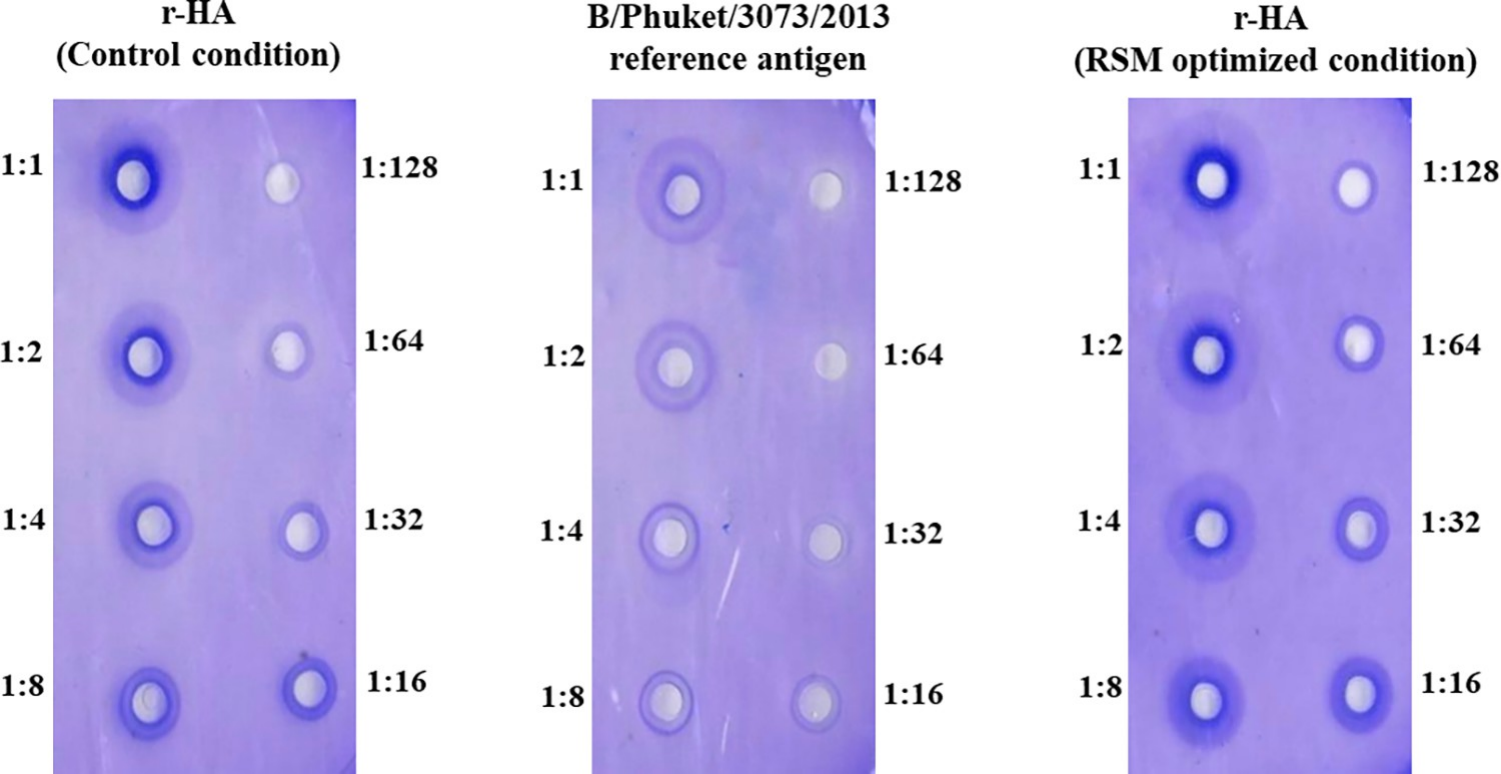
Recombinant HA potency assay in control and RSM-optimized conditions compared to the standard HA antigen.

The potency of r-HA expressed under control and optimized conditions was evaluated through the SRID method (Fig 6) compared to the standard HA protein. Through similar dilution rates (1:1 to 1:128), the potency of r-HA proteins expressed under control and RSM-optimized conditions were 128 and 332 μg/ml, respectively indicating a 2.6-fold increased potency.

### Protein characterization

Following final filtration and formulation, protein characterization of the r-HA drug substance was done for glycosylation pattern, size heterogeneity, native fold, and host cell protein content in comparison to the original product (Flublok®) characteristics by standard and homemade assays (Table 3, S1 Fig).

**Table 3:** Characterization of r-HA drug substance.

Test	Method	Specification	Control condition	Optimized condition
Glycosylation pattern	HILIC following PNGase F treatment	Comparable with standard	Comparable	Comparable
Size heterogeneity	SE-HPLC	Main peak at ~6 ± 1 min	RT: 5.4 min	RT: 5.5 min
			Area: 100%	Area: 100%
Native folding	Trypsin resistance assay	28 and 50 kDa bands in reduced SDS-PAGE	28 and 50kDa bands	28 and 50kDa bands
Host cell protein content	ELISA	NMT 105 ng/μg of HA protein	10 ng/μg	89 ng/μg

## Discussion

According to the literature, different physicochemical and supplemental parameters have been repeatedly reported as potentially important factors in cell culture and protein expression. To our knowledge, optimization of these critical parameters using DOE has not been reported in the case of HA protein expression in Sf9 insect cell culture. Therefore, in the present study, a set of experiments was designed to screen and then optimize the important selected parameters involved in the suspension culture of insect cells through the design of the experiment approach to achieve a higher level of recombinant HA protein expression. Due to ethical, safety, cost, and regulatory concerns [35–37], the selected supplements had non-animal origins.

As previously mentioned, the design of the experiments includes multiple methods such as one factor at a time, full factorial, and fractional factorial design. In a full factorial design, all selected parameters are examined at all levels. This approach is the most comprehensive, but it is also costly and time-consuming [20]. Fractional factorial design (screening) is a type of full factorial design that uses fewer experiments to save time and budget [20]. Plackett-Burman, a two-level classical screening method, identifies critical parameters and their main effects without considering the interactions between studied factors [38]. Response surface methodology (RSM) fits mathematical models to identify the optimal levels of the studied parameters, their combination, and predicts the responses using the obtained equations [20]. The most commonly used approaches in RSM include Box-Behnken design (BBD) and the central composite design (CCD) [39]. BBD, the most efficient RSM approach, provides reliable information through a minimum number of experiments compared to CCD. It requires three levels (-1, 0, 1) for each parameter and can be applied to 3 to 21 numerical and categorical parameters. It aids in detecting nonlinearity and interactions among parameter factors [39]. In CCD, five levels (-α, -1, 0, 1, α as 1.414) will be defined for 2 to 50 parameters. Similar to BBD, CCD can be applied for both numerical and categorical parameters and helps in the detection of nonlinearity and interactions among parameters [39]. Taken together, BBD was selected which defines 3 levels for each parameter.

The initial screening of 12 selected parameters was done by the Placket-Burman approach and 6 selected parameters underwent further optimization to reach the optimized conditions. Polynomial regression and RSM were used in the present study due to the mild nonlinearity among studied variables. Support vector regression (SVR), multiple linear regression, and artificial neural network (ANN) are also powerful theoretical methods based on statistical learning theory which deal with data nonlinearities [40, 41]. The use of machine learning (ML) and artificial intelligence has attracted attention in applied sciences like Biotechnology and Biology. It evaluates suitable and experimental models to identify potential patterns, especially in the case of complex and huge datasets. In the bioprocess development and scale-up procedure of biotechnological products, it is crucial to identify optimal parameters/conditions. Due to the huge number of potentially unknown parameters and processes, the application of ML can help in designing the experiments, predicting the models, and identifying the significant parameters (based on the selected outputs such as cell viability, and protein expression level) in a shorter time at a lower cost [reviewed in 42].

A 5% feeding strategy was observed to have a significant positive effect on HA expression level. Previous studies have shown that during viral infection, nutritional depletion can reduce the expression of recombinant proteins in insect cells [43]. On the other hand, the accumulation of by-products in the culture medium may negatively impact cellular physiology for the expression of recombinant proteins [44]. This phenomenon can be overcome through fed-batch cultivation which significantly enhances the production of desired proteins [10, 45–47].

On the other hand, the significant negative effect of CCI on HA expression level could be due to the nutritional depletion following uncontrolled cell density increase. Therefore, it is important to carefully select the optimum cell density value at the time of the infection and its ratio to the viral load to prevent the lack of nutrients and accumulation of toxic compounds to maximize cellular productivity [22, 44]. Hence, a CCI of 5 ×10^6^ cells/ml was defined as the optimal value to achieve the highest protein expression level. Low MOI values (<1PFU/ml) not only offer economic advantages but also prevent nutritional depletion during the post-infection phase. In contrast, high MOI values (>2 PFU/ml) lead to a synchronous infection which stops cell growth following the infection [22, 23]. Our results demonstrated that MOI values near 1.65 PFU/ml could result in optimal protein expression levels.

The role of temperature, as a physicochemical parameter, was also investigated and a statistically significant effect was found. Previous studies have shown that insect cell growth and its ability to express recombinant proteins can significantly increase by lowering the temperature from 27°C [24, 48–50]. Although the temperature shift had a significant effect in the Placket-Burman screening step, it was not significantly effective on protein expression in the RSM optimization step.

Previous studies have indicated that adding polyamine improves membrane rigidity and prevents lipid oxidation. It results in nucleic acid stabilization and transcription regulation which have positive impact on the production of enveloped viruses [26, 51–53].

Cholesterol is also assumed an external essential additive for insect cell culture which is required for the flexibility of the cell membrane [24, 25]. It also has been reported that adding cholesterol and polyamines together can boost the specific yields of BVs by 7-fold [26]. Therefore, optimization of their concentrations in the culture medium will directly affect the yield of produced viral particles. Although cholesterol (4 mg/L) represented a positive effect in PB-designed experiments, it did not show significant interactions within RSM-designed analyses.

Copolymers like Pluronic are usually recommended for insect cell bioreactor cultures for protection against shear stress of agitation and sparging [22, 54] and their optimum concentration should be reached. Analysis of the Placket-Burman designed experiments revealed that this additive has a significant effect on HA expression, which was not confirmed in RSM. It can be assumed that other parameters, such as the unpublished components of the feed, may neutralize its effect.

Glucose and L-glutamine are the most important sources of carbon and nitrogen that can be metabolized by Sf9 cells. Supplementation of culture medium with different concentrations of these two components has resulted in varied cell growth density, and specific yields of the recombinantly expressed proteins [23, 33, 55–57]. It also has been shown that supplementation of insect cell culture medium with metal ionic salts such as ZnSO_4_ can enhance viral replication and consequently the yield of the recombinant protein up to 100% through reducing the negative charge of the surface of the virus and the insect cell [31]. However, these findings were not supported by the present study, possibly due to their unknown concentrations in the basal medium used.

The cell density was monitored as another main response besides HA expression level. The initial cell density was the only factor affecting this response, reconfirming the importance of the seeding strategy through the Placket-Burman designed experiments for more rapid arrival to the S-phase of the cell cycle [30, 58–61]. Taken together, supplementation of culture medium through selection and optimization steps resulted in approximately 3.36-fold increased r-HA expression level. Although our results reconfirmed the success of traditional DOE approaches using ANOVA regression analysis in the optimization of parameters, it is worth noting that newly developed methods such as artificial intelligence, machine learning (ML), and global sensitivity analysis (using SOBOL’ sampling method) should get more attention for a better and deeper understanding of the effective uncertain parameters [62].

Due to the importance of the scale-up procedure in eukaryotic cell cultures and because of cell sensitivity to the shear stress [63, 64], we conducted a scale-up process, shifting from laboratory 500 ml shake flasks (working volume of 100 ml) to 2L bioreactor systems under RSM-optimized condition in which reproducibility of the procedure and r-HA expression level were reconfirmed. The SRID potency assay revealed that r-HA protein expressed under RSM-optimized conditions is 2.6-fold more potent than HA protein produced under control conditions. This improvement could be due to the optimization and supplementation of the cell culture medium. Further characterization studies represented comparable results of r-HA protein expressed under un-supplemented and optimized conditions when the original commercially available recombinant protein was used as the reference protein. The observed comparability revealed that manipulation of culture conditions and its supplementation had no negative effect on the qualitative properties of the protein.

## Conclusions

Our study showed that the design of experiments (DOE) resulted in increased r-HA protein expression and potency compared to the control condition where supplementation of culture medium did not occur. Due to the importance of HA potency for its application, any increase in potency would reduce the amount of protein required for filling, leading to cost reduction in industrial manufacturing. Besides, an important issue in optimization studies is the identification of effective parameters for sensitivity and uncertainty analysis to ensure the feasibility and robustness of the studies, which should be considered in the next complementary experiments.

## Supporting information

S1 Table Plackett-Burman experimental design. (DOCX)

S2 Table Box-Behnken designed experiments. (DOCX)

S3 Table Daily effect of studied parameters on A) viability, B) viable cell count within 23 Placket-Burman-designed experiments. The gray cells do not have data due to the early harvesting.
(DOCX)

S4 Table Effect of studied parameters on A) Viable cell count, B) Viability within designed 54 experiments. The gray cells do not have data due to the early harvesting.
(DOCX)

S1 Fig Characterization of r-HA drug substance. **Glycosylation pattern: (A)** Reference HA protein, **(B)** r-HA produced under control condition, **(C)** r-HA produced under optimized condition. **Size Heterogeneity: (D)** Reference HA protein, **(E)** r-HA produced under control condition, **(F)** r-HA produced under optimized condition. **Native folding (reduced SDS-PAGE): (A)** Reference HA protein; **(B)** r-HA produced under control condition; **(C)** r-HA produced under optimized condition: #1, 6, 9: HA protein treated with high concentration of Trypsin (1050 μg/ml); #2, 5, 8: HA protein treated with low concentration of Trypsin (210 μg/ml); # 3, 4, 7: HA protein without Trypsin digestion.
(DOCX)
