# Supplementary material for: Optimization of culture condition for Spodoptera frugiperda by design of experiment approach and evaluation of its effect on the expression of hemagglutinin protein of influenza virus
Source: PLoS One. 2024 Aug 16;19(8):e0308547. doi: 10.1371/journal.pone.0308547 (PMC11329130; doi:10.1371/journal.pone.0308547)
Supplement: S1 Table — (DOCX) [file pone.0308547.s001.docx]

| **Runs** | **Feed (%)** | **CCI** | **MOI** | **Initial cell density** | **Temperature** | **Cholesterol** | **Polyamine** | **Galactose** | **Pluronic** | **Glucose** | **L-glutamine** | **ZnSO_4_** |
| --- | --- | --- | --- | --- | --- | --- | --- | --- | --- | --- | --- | --- |
| 1 | 0 | 0 | 0 | 0 | 0 | 0 | 0 | 0 | 0 | 0 | 0 | 0 |
| 2 | -1 | 1 | -1 | -1 | -1 | -1 | -1 | 1 | 1 | 1 | 1 | -1 |
| 3 | -1 | -1 | -1 | 1 | -1 | 1 | 1 | -1 | 1 | -1 | -1 | 1 |
| 4 | 1 | 1 | -1 | -1 | -1 | 1 | 1 | 1 | 1 | 1 | -1 | 1 |
| 5 | -1 | -1 | 1 | -1 | 1 | 1 | 1 | -1 | 1 | 1 | -1 | -1 |
| 6 | 1 | 1 | 1 | -1 | 1 | -1 | 1 | -1 | 1 | -1 | -1 | -1 |
| 7 | -1 | 1 | 1 | 1 | -1 | 1 | 1 | -1 | -1 | 1 | 1 | 1 |
| 8 | 1 | -1 | -1 | 1 | 1 | 1 | 1 | 1 | -1 | -1 | 1 | -1 |
| 9 | 1 | 1 | -1 | 1 | 1 | 1 | -1 | -1 | 1 | 1 | 1 | -1 |
| 10 | 1 | -1 | -1 | 1 | -1 | -1 | 1 | 1 | -1 | 1 | -1 | -1 |
| 11 | 1 | -1 | 1 | 1 | 1 | -1 | -1 | 1 | 1 | 1 | -1 | 1 |
| 12 | -1 | 1 | 1 | 1 | -1 | -1 | 1 | 1 | 1 | -1 | 1 | -1 |
| 13 | -1 | 1 | -1 | 1 | 1 | -1 | -1 | -1 | -1 | 1 | -1 | 1 |
| 14 | -1 | -1 | -1 | -1 | -1 | -1 | -1 | -1 | -1 | -1 | -1 | -1 |
| 15 | 1 | 1 | 1 | -1 | -1 | 1 | -1 | 1 | -1 | -1 | -1 | 1 |
| 16 | -1 | -1 | -1 | -1 | 1 | 1 | -1 | 1 | 1 | -1 | 1 | 1 |
| 17 | 1 | 1 | -1 | -1 | 1 | -1 | 1 | -1 | -1 | -1 | 1 | 1 |
| 18 | 0 | 0 | 0 | 0 | 0 | 0 | 0 | 0 | 0 | 0 | 0 | 0 |
| 19 | -1 | 1 | 1 | 1 | 1 | 1 | -1 | 1 | -1 | -1 | -1 | -1 |
| 20 | 1 | -1 | 1 | 1 | -1 | -1 | -1 | -1 | 1 | -1 | 1 | 1 |
| 21 | 1 | -1 | 1 | -1 | -1 | 1 | -1 | -1 | -1 | 1 | 1 | -1 |
| 22 | 0 | 0 | 0 | 0 | 0 | 0 | 0 | 0 | 0 | 0 | 0 | 0 |
| 23 | -1 | -1 | 1 | -1 | 1 | -1 | 1 | 1 | -1 | 1 | 1 | 1 |
