# Supplementary material for: Optimization of culture condition for Spodoptera frugiperda by design of experiment approach and evaluation of its effect on the expression of hemagglutinin protein of influenza virus
Source: PLoS One. 2024 Aug 16;19(8):e0308547. doi: 10.1371/journal.pone.0308547 (PMC11329130; doi:10.1371/journal.pone.0308547)
Supplement: S2 Table — (DOCX) [file pone.0308547.s002.docx]

| **Runs** | **Feed**  **percentage** | **CCI** | **MOI** | **Temperature** | **Cholesterol** | **Pluronic** | **Runs** | **Feed**  **percentage** | **CCI** | **MOI** | **Temperature** | **Cholesterol** | **Pluronic** |
| --- | --- | --- | --- | --- | --- | --- | --- | --- | --- | --- | --- | --- | --- |
| **1** | 0 | 0 | -1 | -1 | 0 | -1 | **28** | -1 | -1 | 0 | 1 | 0 | 0 |
| **2** | 0 | 0 | 0 | 0 | 0 | 0 | **29** | 0 | 0 | -1 | -1 | 0 | 1 |
| **3** | 0 | 0 | -1 | 1 | 0 | 1 | **30** | 0 | 1 | 1 | 0 | 1 | 0 |
| **4** | -1 | 1 | 0 | 1 | 0 | 0 | **31** | 1 | 0 | 1 | 0 | 0 | 1 |
| **5** | -1 | 0 | 1 | 0 | 0 | 1 | **32** | 0 | -1 | 0 | 0 | -1 | 1 |
| **6** | 0 | 1 | 0 | 0 | -1 | 1 | **33** | 0 | 1 | 0 | 0 | 1 | 1 |
| **7** | 0 | 1 | 0 | 0 | 1 | -1 | **34** | -1 | 0 | 0 | -1 | 1 | 0 |
| **8** | 1 | 1 | 0 | -1 | 0 | 0 | **35** | 1 | 0 | 0 | 1 | 1 | 0 |
| **9** | 1 | 0 | 0 | -1 | 1 | 0 | **36** | 0 | -1 | 1 | 0 | -1 | 0 |
| **10** | 0 | 1 | 1 | 0 | -1 | 0 | **37** | -1 | 0 | 1 | 0 | 0 | -1 |
| **11** | 0 | 1 | -1 | 0 | 1 | 0 | **38** | 0 | -1 | -1 | 0 | 1 | 0 |
| **12** | 0 | -1 | 0 | 0 | 1 | 1 | **39** | 0 | 0 | 1 | -1 | 0 | -1 |
| **13** | 0 | 0 | 0 | 0 | 0 | 0 | **40** | 1 | 0 | -1 | 0 | 0 | -1 |
| **14** | 1 | 0 | -1 | 0 | 0 | 1 | **41** | 0 | 1 | 0 | 0 | -1 | -1 |
| **15** | 0 | 0 | 1 | 1 | 0 | -1 | **42** | -1 | 0 | 0 | 1 | -1 | 0 |
| **16** | 0 | 0 | 1 | -1 | 0 | 1 | **43** | 0 | 1 | -1 | 0 | -1 | 0 |
| **17** | 0 | -1 | 1 | 0 | 1 | 0 | **44** | 0 | 0 | -1 | 1 | 0 | -1 |
| **18** | 0 | 0 | 0 | 0 | 0 | 0 | **45** | 0 | -1 | 0 | 0 | 1 | -1 |
| **19** | -1 | -1 | 0 | -1 | 0 | 0 | **46** | 0 | 0 | 0 | 0 | 0 | 0 |
| **20** | 1 | 0 | 1 | 0 | 0 | -1 | **47** | 1 | 0 | 0 | -1 | -1 | 0 |
| **21** | 1 | 0 | 0 | 1 | -1 | 0 | **48** | 0 | 0 | 0 | 0 | 0 | 0 |
| **22** | -1 | 0 | 0 | 1 | 1 | 0 | **49** | 0 | 0 | 1 | 1 | 0 | 1 |
| **23** | 0 | -1 | -1 | 0 | -1 | 0 | **50** | 0 | 0 | 0 | 0 | 0 | 0 |
| **24** | 0 | -1 | 0 | 0 | -1 | -1 | **51** | 1 | -1 | 0 | -1 | 0 | 0 |
| **25** | -1 | 0 | 0 | -1 | -1 | 0 | **52** | -1 | 1 | 0 | -1 | 0 | 0 |
| **26** | -1 | 0 | -1 | 0 | 0 | -1 | **53** | 1 | 1 | 0 | 1 | 0 | 0 |
| **27** | 1 | -1 | 0 | 1 | 0 | 0 | **54** | -1 | 0 | -1 | 0 | 0 | 1 |
