# Supplementary material for: Optimization of culture condition for Spodoptera frugiperda by design of experiment approach and evaluation of its effect on the expression of hemagglutinin protein of influenza virus
Source: PLoS One. 2024 Aug 16;19(8):e0308547. doi: 10.1371/journal.pone.0308547 (PMC11329130; doi:10.1371/journal.pone.0308547)
Supplement: S3 Table — Daily effect of studied parameters on A) viability, B) viable cell count within 23 Placket-Burman-designed experiments. The gray cells do not have data due to the early harvesting. (DOCX) [file pone.0308547.s003.docx]

|  | 1. **Viability (%)** | | | | | | | | | | | | | | | | | | | | | | |
| --- | --- | --- | --- | --- | --- | --- | --- | --- | --- | --- | --- | --- | --- | --- | --- | --- | --- | --- | --- | --- | --- | --- | --- |
| **Days** | **1** | **2** | **3** | **4** | **5** | **6** | **7** | **8** | **9** | **10** | **11** | **12** | **13** | **14** | **15** | **16** | **17** | **18** | **19** | **20** | **21** | **22** | **23** |
| **0** | 98.6 | 98.8 | 98.5 | 98.8 | 98.8 | 98.8 | 98.5 | 98.5 | 98.5 | 98.5 | 98.5 | 98.5 | 98.5 | 98.8 | 98.8 | 98.8 | 98.8 | 98.6 | 98.5 | 98.5 | 98.8 | 98.6 | 98.8 |
| **1** | 99.0 | 98.9 | 96.2 | 98.7 | 96.7 | 97.0 | 97.2 | 97.1 | 98.9 | 98.3 | 97.2 | 96.0 | 96.2 | 96.5 | 96.4 | 97.7 | 99.6 | 98.7 | 97.0 | 96.4 | 97.7 | 94.7 | 95.7 |
| **2** | 96.8 | 94.0 | 96.5 | 95.9 | 94.4 | 96.2 | 94.1 | 95.6 | 94.7 | 94.5 | 94.1 | 96.2 | 95.3 | 95.1 | 96.0 | 93.2 | 94.9 | 96.8 | 97.6 | 94.6 | 91.9 | 91.9 | 92.7 |
| **3** | 98.2 | 99.4 | 98.4 | 98.2 | 97.6 | 99.0 | 98.3 | 97.9 | 97.8 | 96.3 | 97.8 | 97.1 | 97.7 | 97.2 | 96.6 | 98.7 | 97.8 | 97.3 | 98.2 | 98.6 | 98.1 | 97.2 | 96.4 |
| **4** | 95.0 | 98.8 | 98.4 | 97.8 | 97.5 | 96.6 | 98.2 | 96.8 | 97.6 | 97.3 | 95.1 | 96.2 | 95.2 | 97.1 | 95.0 | 97.0 | 92.6 | 98.6 | 97.9 | 98.9 | 95.2 | 97.4 | 94.6 |
| **5** | 85.9 | 98.1 | 91.8 | 95.4 | 50.9 | 93.9 | 95.7 | 80.0 | 87.7 | 95.6 | 53.3 | 95.0 | 87.9 | 92.4 | 95.0 | 73.1 | 89.0 | 76.7 | 89.9 | 90.9 | 88.8 | 71.1 | 47.1 |
| **6** | 54.3 | 93.3 | 61.3 | 88.1 |  | 81.4 | 82.6 | 39.0 | 70.9 | 73.7 |  | 87.3 | 74.2 | 67.2 | 84.2 | 47.7 | 78.8 | 53.1 | 79.2 | 57.1 | 56.9 | 55.0 |  |
| **7** |  | 86.3 |  | 68.8 |  | 52.0 | 63.8 |  | 56.2 | 50.0 |  | 66.7 | 64.0 | 50.0 | 62.9 |  | 41.1 |  | 67.8 |  |  |  |  |
| 1. **Viable cell count (×10^6^ cells/ml)** | | | | | | | | | | | | | | | | | | | | | | | |
| **Days** | **1** | **2** | **3** | **4** | **5** | **6** | **7** | **8** | **9** | **10** | **11** | **12** | **13** | **14** | **15** | **16** | **17** | **18** | **19** | **20** | **21** | **22** | **23** |
| **0** | 1.4 | 0.8 | 2.0 | 0.8 | 0.8 | 0.8 | 2.0 | 2.0 | 2.0 | 2.0 | 2.0 | 2.0 | 2.0 | 0.8 | 0.8 | 0.8 | 0.8 | 1.4 | 2.0 | 2.0 | 0.8 | 1.4 | 0.8 |
| **1** | 3.0 | 1.8 | 4.0 | 1.5 | 1.5 | 1.6 | 4.1 | 4.3 | 4.3 | 4.7 | 3.8 | 4.1 | 4.5 | 1.6 | 1.6 | 2.2 | 2.3 | 3.8 | 3.8 | 3.5 | 1.7 | 2.8 | 1.6 |
| **2** | 5.4 | 2.8 | 6.0 | 2.8 | 2.7 | 3.0 | 6.1 | 6.1 | 6.4 | 6.5 | 6.2 | 5.6 | 5.7 | 2.9 | 3.4 | 2.3 | 2.6 | 5.4 | 4.9 | 5.9 | 2.5 | 4.3 | 2.8 |
| **3** | 7.4 | 6.4 | 7.2 | 4.9 | 4.9 | 6.1 | 6.4 | 8.0 | 7.4 | 7.5 | 6.8 | 6.0 | 6.7 | 5.5 | 5.7 | 6.1 | 5.4 | 6.9 | 6.5 | 7.6 | 5.8 | 7.0 | 4.6 |
| **4** | 3.8 | 5.1 | 1.9 | 5.7 | 2.4 | 4.9 | 4.3 | 2.4 | 6.1 | 2.2 | 2.7 | 4.8 | 5.8 | 2.7 | 5.3 | 1.9 | 7.5 | 4.3 | 3.8 | 1.8 | 2.0 | 4.6 | 2.4 |
| **5** | 3.4 | 6.3 | 1.8 | 7.1 | 1.1 | 5.4 | 4.4 | 2.1 | 6.4 | 2.4 | 1.0 | 4.6 | 5.3 | 3.9 | 5.1 | 1.9 | 5.8 | 3.0 | 4.7 | 2.6 | 1.9 | 3.2 | 1.2 |
| **6** | 1.9 | 5.6 | 1.4 | 4.8 |  | 4.6 | 3.6 | 0.8 | 4.0 | 1.6 |  | 4.0 | 4.9 | 1.8 | 4.9 | 1.2 | 6.7 | 2.0 | 3.5 | 1.3 | 1.2 | 2.4 |  |
| **7** |  | 5.8 |  | 4.4 |  | 3.2 | 3.0 |  | 3.3 | 1.4 |  | 3.0 | 4.4 | 2.2 | 4.4 |  | 3.0 |  | 3.2 |  |  |  |  |
