# Supplementary material for: Optimization of culture condition for Spodoptera frugiperda by design of experiment approach and evaluation of its effect on the expression of hemagglutinin protein of influenza virus
Source: PLoS One. 2024 Aug 16;19(8):e0308547. doi: 10.1371/journal.pone.0308547 (PMC11329130; doi:10.1371/journal.pone.0308547)
Supplement: S4 Table — Effect of studied parameters on A) Viable cell count, B) Viability within designed 54 experiments. The gray cells do not have data due to the early harvesting. (DOCX) [file pone.0308547.s004.docx]

|  | 1. **Viable cell count (×10^6^ cells/ml)** | | | | | | | | | | | | | | | | | | | | | | | | | | |
| --- | --- | --- | --- | --- | --- | --- | --- | --- | --- | --- | --- | --- | --- | --- | --- | --- | --- | --- | --- | --- | --- | --- | --- | --- | --- | --- | --- |
| **Days** | **1** | **2** | **3** | **4** | **5** | **6** | **7** | **8** | **9** | **10** | **11** | **12** | **13** | **14** | **15** | **16** | **17** | **18** | **19** | **20** | **21** | **22** | **23** | **24** | **25** | **26** | **27** |
| **0** | 1.2 | 1.2 | 1.2 | 1.2 | 1.2 | 1.2 | 1.2 | 1.2 | 1.2 | 1.2 | 1.2 | 1.2 | 1.2 | 1.2 | 1.2 | 1.2 | 1.2 | 1.2 | 1.2 | 1.2 | 1.2 | 1.2 | 1.2 | 1.2 | 1.2 | 1.2 | 1.2 |
| **1** | 2.0 | 2.0 | 2.1 | 2.1 | 2.0 | 2.0 | 2.1 | 2.0 | 2.0 | 2.0 | 2.1 | 2.0 | 2.1 | 2.2 | 2.1 | 2.2 | 2.2 | 2.0 | 2.2 | 2.1 | 2.1 | 2.2 | 2.0 | 2.1 | 2.2 | 2.0 | 2.2 |
| **2** | 4.6 | 5.2 | 5.1 | 5.2 | 5.0 | 4.6 | 4.9 | 5.0 | 4.6 | 5.4 | 4.7 | 4.7 | 4.6 | 5.1 | 4.6 | 5.3 | 5.2 | 4.0 | 5.2 | 4.7 | 4.4 | 5.0 | 4.8 | 4.5 | 5.2 | 4.0 | 5.3 |
| **3** | 8.0 | 6.6 | 7.6 | 6.5 | 7.0 | 6.9 | 7.1 | 8.4 | 7.7 | 7.0 | 7.2 | 6.6 | 7.7 | 7.4 | 7.6 | 7.0 | 7.0 | 7.6 | 7.1 | 7.6 | 7.0 | 6.3 | 6.5 | 7.8 | 6.6 | 6.9 | 7.4 |
| **4** | 4.7 | 4.0 | 5.9 | 5.4 | 3.4 | 6.1 | 6.2 | 5.8 | 4.0 | 5.8 | 6.8 | 2.3 | 4.2 | 5.1 | 3.8 | 4.1 | 2.7 | 3.9 | 2.0 | 3.8 | 5.4 | 3.7 | 2.5 | 2.1 | 4.1 | 5.0 | 2.1 |
| **5** | 4.7 | 4.2 | 4.4 | 4.4 | 3.8 | 5.7 | 6.5 | 5.4 | 4.4 | 5.4 | 7.0 | 2.6 | 3.0 | 5.9 | 2.4 | 4.2 | 2.7 | 3.6 | 2.4 | 3.3 | 2.6 | 3.3 | 2.1 | 2.3 | 3.5 | 4.5 | 1.1 |
| **6** | 3.9 | 2.8 | 2.6 | 1.4 | 2.2 | 4.2 | 4.2 | 3.8 | 2.3 | 4.2 | 6.0 | 1.2 | 2.5 | 4.2 |  | 1.6 | 1.3 | 1.7 | 1.0 | 2.1 |  | 1.8 | 1.4 | 1.0 | 1.8 | 4.6 |  |
| **7** | 3.5 | 2.1 |  |  |  | 3.8 | 3.0 |  |  | 2.5 | 4.8 |  |  | 3.2 |  |  |  |  |  |  |  |  |  |  |  | 3.2 |  |
| **Days** | **28** | **29** | **30** | **31** | **32** | **33** | **34** | **35** | **36** | **37** | **38** | **39** | **40** | **41** | **42** | **43** | **44** | **45** | **46** | **47** | **48** | **49** | **50** | **51** | **52** | **53** | **54** |
| **0** | 1.2 | 1.2 | 1.2 | 1.2 | 1.2 | 1.2 | 1.2 | 1.2 | 1.2 | 1.2 | 1.2 | 1.2 | 1.2 | 1.2 | 1.2 | 1.2 | 1.2 | 1.2 | 1.2 | 1.2 | 1.2 | 1.2 | 1.2 | 1.2 | 1.2 | 1.2 | 1.2 |
| **1** | 2.0 | 2.0 | 2.5 | 2.0 | 2.2 | 2.0 | 2.1 | 1.8 | 2.6 | 2.2 | 2.3 | 2.2 | 2.7 | 1.9 | 2.5 | 2.3 | 2.1 | 1.8 | 1.9 | 1.8 | 1.9 | 2.7 | 2.0 | 2.2 | 1.6 | 1.8 | 2.5 |
| **2** | 4.6 | 4.0 | 4.0 | 3.7 | 4.4 | 3.9 | 3.5 | 4.0 | 4.5 | 4.8 | 3.8 | 4.3 | 3.6 | 4.2 | 3.8 | 3.8 | 4.0 | 5.0 | 4.0 | 3.8 | 4.7 | 4.0 | 4.2 | 4.2 | 4.2 | 4.3 | 4.0 |
| **3** | 6.4 | 6.7 | 7.8 | 7.0 | 7.2 | 7.2 | 6.8 | 7.6 | 7.1 | 8.0 | 6.8 | 8.0 | 6.6 | 6.9 | 6.6 | 6.7 | 7.6 | 6.7 | 7.2 | 6.6 | 7.0 | 6.0 | 7.6 | 7.0 | 7.4 | 6.4 | 7.1 |
| **4** | 2.4 | 6.0 | 6.0 | 4.6 | 2.0 | 5.7 | 3.8 | 4.4 | 2.2 | 3.6 | 2.8 | 3.6 | 6.8 | 6.4 | 4.3 | 6.0 | 5.8 | 2.3 | 4.9 | 5.6 | 4.3 | 5.0 | 3.0 | 2.3 | 5.7 | 6.5 | 4.0 |
| **5** | 1.7 | 5.5 | 5.2 | 5.0 | 1.9 | 5.8 | 3.7 | 2.5 | 2.2 | 3.9 | 2.7 | 3.4 | 6.0 | 6.0 | 2.7 | 7.2 | 3.8 | 2.8 | 4.1 | 5.6 | 4.3 | 4.2 | 3.2 | 2.6 | 5.6 | 6.8 | 4.1 |
| **6** | 0.5 | 5.2 | 4.9 | 3.2 | 1.1 | 4.8 | 3.2 |  | 1.1 | 2.0 | 1.4 | 2.4 | 6.0 | 5.0 |  | 5.5 | 2.4 | 1.1 | 1.9 | 3.7 | 3.0 | 2.6 | 1.6 | 1.3 | 5.0 | 3.2 | 3.1 |
| **7** |  | 4.9 | 3.1 |  |  | 3.4 | 3.0 |  |  |  |  |  | 5.4 | 3.6 |  | 5.0 |  |  |  |  |  |  |  |  | 4.0 |  | 2.8 |
|  | 1. **Viability (%)** | | | | | | | | | | | | | | | | | | | | | | | | | | |
| **Days** | **1** | **2** | **3** | **4** | **5** | **6** | **7** | **8** | **9** | **10** | **11** | **12** | **13** | **14** | **15** | **16** | **17** | **18** | **19** | **20** | **21** | **22** | **23** | **24** | **25** | **26** | **27** |
| **0** | 96.0 | 96.0 | 96.0 | 96.0 | 96.0 | 96.0 | 96.0 | 96.0 | 96.0 | 96.0 | 96.0 | 96.0 | 96.0 | 96.0 | 96.0 | 96.0 | 96.0 | 96.0 | 96.0 | 96.0 | 96.0 | 96.0 | 96.0 | 96.0 | 96.0 | 96.0 | 96.0 |
| **1** | 95.2 | 96.2 | 96.7 | 97.7 | 97.6 | 96.6 | 97.7 | 97.1 | 95.2 | 96.6 | 97.7 | 96.2 | 96.3 | 97.8 | 97.5 | 96.5 | 96.5 | 95.2 | 96.5 | 97.7 | 95.5 | 96.9 | 95.2 | 96.8 | 97.8 | 96.2 | 97.8 |
| **2** | 98.9 | 98.3 | 99.2 | 98.5 | 97.3 | 97.7 | 97.8 | 99.4 | 95.4 | 98.2 | 98.1 | 96.7 | 96.4 | 98.6 | 97.0 | 97.1 | 98.1 | 95.5 | 96.7 | 98.5 | 95.7 | 97.3 | 94.5 | 96.4 | 98.9 | 97.6 | 98.3 |
| **3** | 99.4 | 99.4 | 98.6 | 98.8 | 98.0 | 98.9 | 98.5 | 98.6 | 99.5 | 98.5 | 98.6 | 97.9 | 99.0 | 98.7 | 98.8 | 99.0 | 98.6 | 98.4 | 97.8 | 98.7 | 98.2 | 99.1 | 98.8 | 98.7 | 97.1 | 99.0 | 98.9 |
| **4** | 97.3 | 97.1 | 95.2 | 96.1 | 97.4 | 98.5 | 98.4 | 95.9 | 98.3 | 98.5 | 98.7 | 98.3 | 98.1 | 97.1 | 95.0 | 96.7 | 99.3 | 97.3 | 97.5 | 94.8 | 92.8 | 93.9 | 97.7 | 98.1 | 95.8 | 97.5 | 94.2 |
| **5** | 97.5 | 90.6 | 85.3 | 88.0 | 89.4 | 96.6 | 98.0 | 94.7 | 90.9 | 97.8 | 97.6 | 94.5 | 83.1 | 95.6 | 55.8 | 91.3 | 92.8 | 85.3 | 89.6 | 90.4 | 57.0 | 83.1 | 92.5 | 90.6 | 89.3 | 92.6 | 54.4 |
| **6** | 72.2 | 70.0 | 60.5 | 48.3 | 56.4 | 83.3 | 84.7 | 64.4 | 53.5 | 85.4 | 92.3 | 53.1 | 58.1 | 72.4 |  | 43.2 | 52.0 | 45.9 | 48.4 | 51.2 |  | 46.2 | 43.1 | 45.5 | 48.6 | 81.6 |  |
| **7** | 64.8 | 43.8 |  |  |  | 72.4 | 71.8 |  |  | 55.6 | 77.4 |  |  | 59.3 |  |  |  |  |  |  |  |  |  |  |  | 66.7 |  |
| **Days** | **28** | **29** | **30** | **31** | **32** | **33** | **34** | **35** | **36** | **37** | **38** | **39** | **40** | **41** | **42** | **43** | **44** | **45** | **46** | **47** | **48** | **49** | **50** | **51** | **52** | **53** | **54** |
| **0** | 96.8 | 96.8 | 96.8 | 96.8 | 96.8 | 96.8 | 96.8 | 96.8 | 96.8 | 96.8 | 96.8 | 96.8 | 96.8 | 96.8 | 96.8 | 96.8 | 96.8 | 96.8 | 96.8 | 96.8 | 96.8 | 96.8 | 96.8 | 96.8 | 96.8 | 96.8 | 96.8 |
| **1** | 97.1 | 95.7 | 96.5 | 96.6 | 96.9 | 97.6 | 95.5 | 97.8 | 98.5 | 94.4 | 95.4 | 95.2 | 97.1 | 95.0 | 96.2 | 97.9 | 96.8 | 96.3 | 96.9 | 97.8 | 92.7 | 98.2 | 98.0 | 96.5 | 96.4 | 95.2 | 94.7 |
| **2** | 97.7 | 98.3 | 97.8 | 97.4 | 98.2 | 97.0 | 96.4 | 95.9 | 98.7 | 98.0 | 97.7 | 98.2 | 97.0 | 96.8 | 97.9 | 98.7 | 97.3 | 98.0 | 98.8 | 97.2 | 97.7 | 98.5 | 98.1 | 99.3 | 97.2 | 98.9 | 95.9 |
| **3** | 98.5 | 98.1 | 98.5 | 97.9 | 98.8 | 98.8 | 97.4 | 97.6 | 99.3 | 98.9 | 97.7 | 98.4 | 97.8 | 97.5 | 98.4 | 98.8 | 97.1 | 97.4 | 97.8 | 96.4 | 98.6 | 97.7 | 98.6 | 97.8 | 97.4 | 97.0 | 98.3 |
| **4** | 92.3 | 96.3 | 98.4 | 97.5 | 97.6 | 97.6 | 94.8 | 95.4 | 97.8 | 92.8 | 98.2 | 90.9 | 97.4 | 97.9 | 90.0 | 96.8 | 97.3 | 91.3 | 97.0 | 94.0 | 94.5 | 91.2 | 92.0 | 94.3 | 95.3 | 96.2 | 95.5 |
| **5** | 66.2 | 96.3 | 96.3 | 85.6 | 87.2 | 97.6 | 89.8 | 52.1 | 90.9 | 85.9 | 98.2 | 82.5 | 96.3 | 90.9 | 58.7 | 95.4 | 77.6 | 66.4 | 84.4 | 90.0 | 85.7 | 73.7 | 88.4 | 70.3 | 97.2 | 92.5 | 97.4 |
| **6** | 31.0 | 86.7 | 86.0 | 61.5 | 45.8 | 84.2 | 78.0 |  | 44.0 | 50.0 | 50.0 | 60.0 | 93.3 | 83.3 |  | 85.9 | 55.8 | 47.8 | 47.5 | 60.7 | 58.8 | 53.1 | 43.2 | 43.3 | 89.3 | 54.2 | 79.5 |
| **7** |  | 83.1 | 62.0 |  |  | 79.1 | 68.2 |  |  |  |  |  | 75.0 | 62.1 |  | 80.6 |  |  |  |  |  |  |  |  | 83.3 |  | 70.0 |
