# Supplementary figures and images for: Optimization of culture condition for Spodoptera frugiperda by design of experiment approach and evaluation of its effect on the expression of hemagglutinin protein of influenza virus
Source: PLoS One. 2024 Aug 16;19(8):e0308547. doi: 10.1371/journal.pone.0308547 (PMC11329130; doi:10.1371/journal.pone.0308547)

| **Glycosylation pattern** | |
| --- | --- |
| **A**  **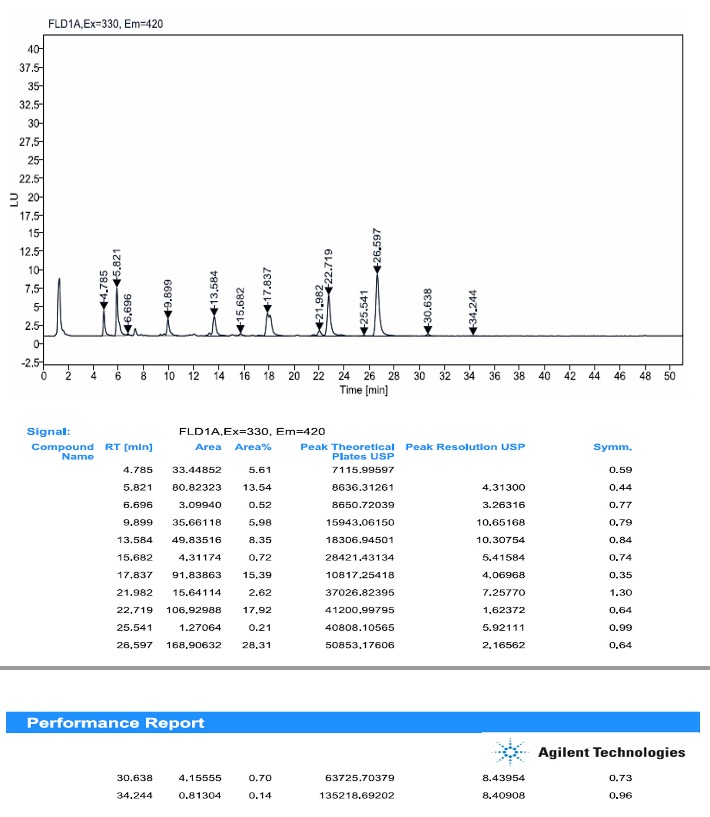** | **B**  **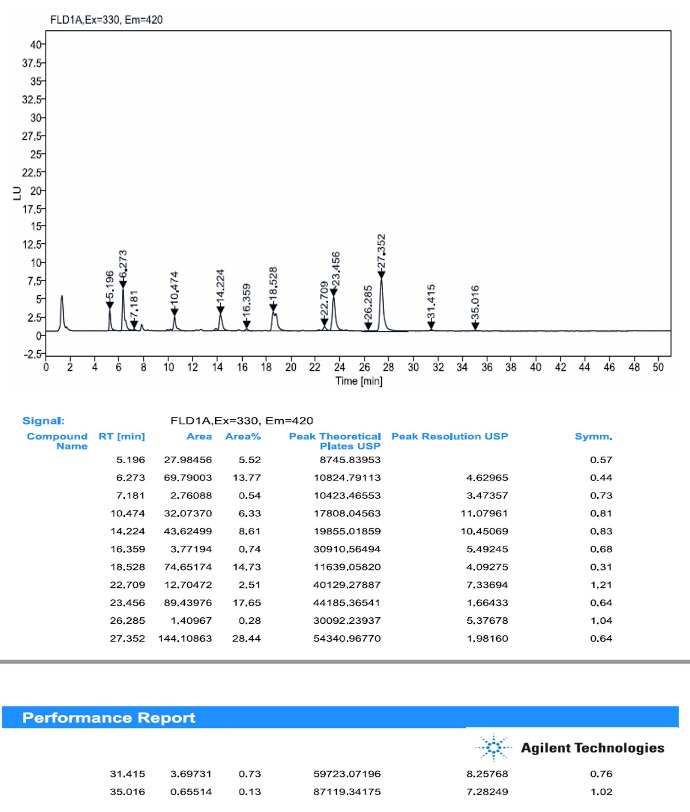** |
| **C**  **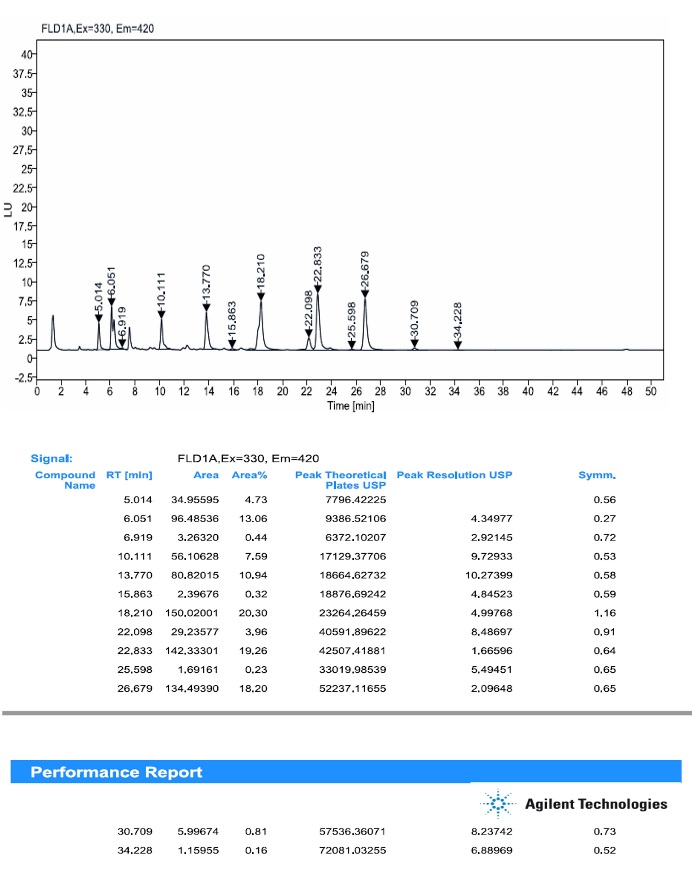** |  |
| **Size Heterogeneity** | |
| **D**  **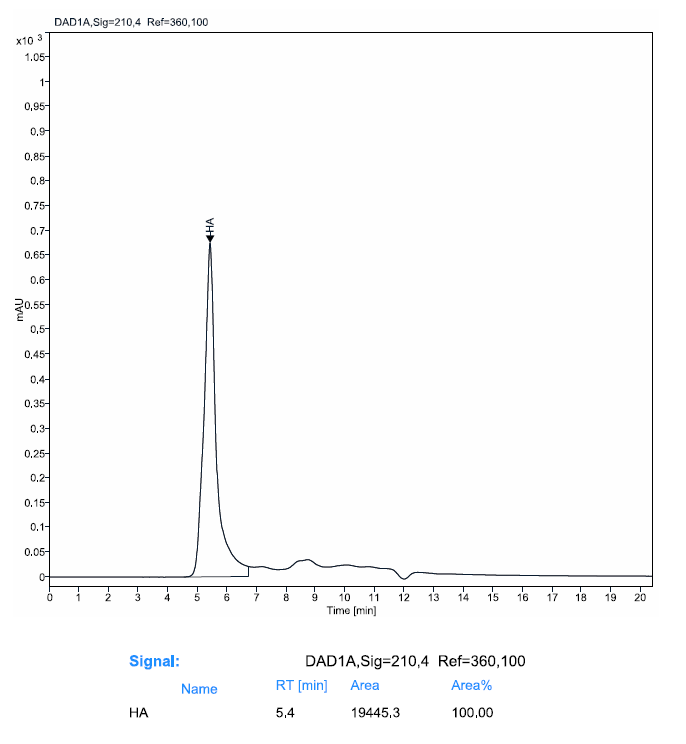** | **E**  **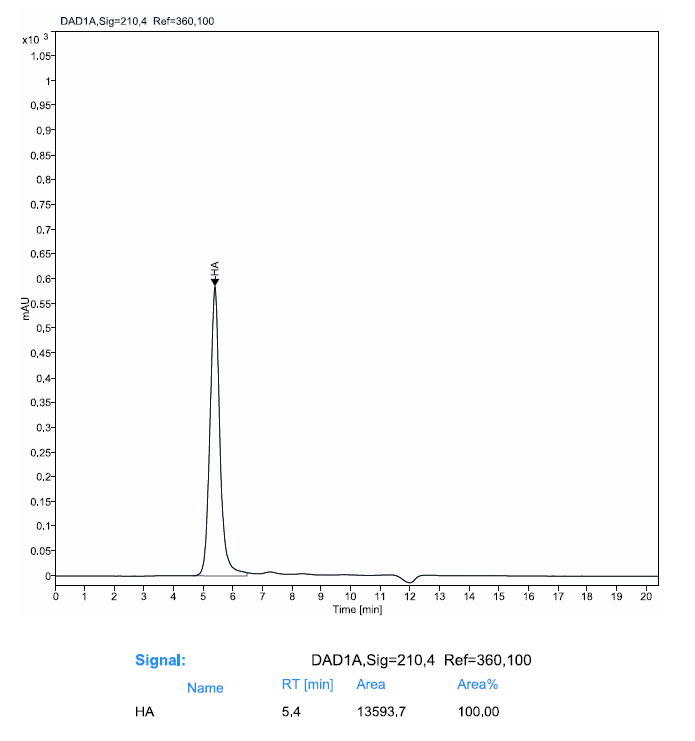** |
| **F**  **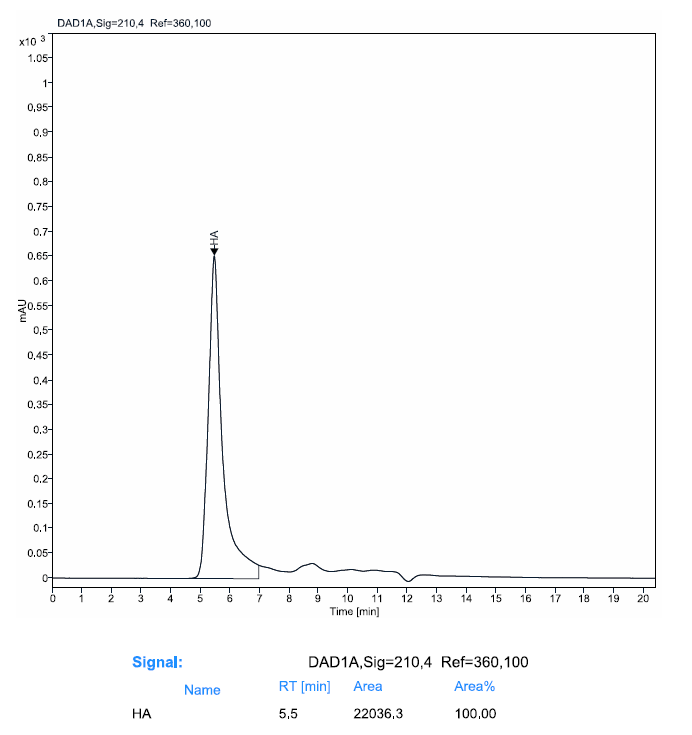** |  |
| **Native folding** | |
| **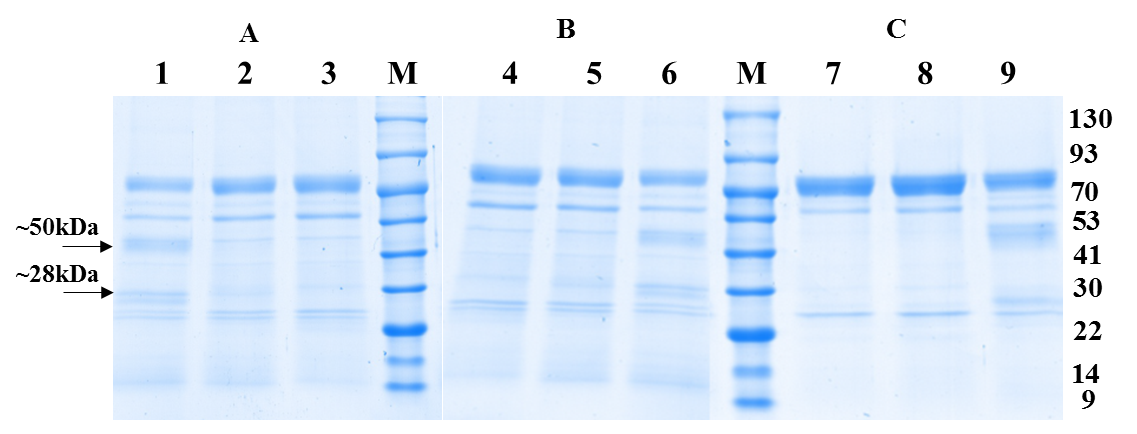** | |

Supplement: S1 Fig — Glycosylation pattern: (A) Reference HA protein, (B) r-HA produced under control condition, (C) r-HA produced under optimized condition. Size Heterogeneity: (D) Reference HA protein, (E) r-HA produced under control condition, (F) r-HA produced under optimized condition. Native folding (reduced SDS-PAGE): (A) Reference HA protein; (B) r-HA produced under control condition; (C) r-HA produced under optimized condition: #1, 6, 9: HA protein treated with high concentration of Trypsin (1050 μg/ml); #2, 5, 8: HA protein treated with low concentration of Trypsin (210 μg/ml); # 3, 4, 7: HA protein without Trypsin digestion. (DOCX) [file pone.0308547.s005.docx]
